# Experimentally-Induced Metabolic Acidosis Does not Alter Aortic Fatty Streak Formation in High-Cholesterol Fed Rabbits

**Published:** 2012

**Authors:** Majid Khazaei, Mehdi Nematbakhsh

**Affiliations:** 1 *Department of Physiology; Isfahan University of Medical Sciences, Isfahan, Iran*; 2 *Water and Electrolytes Research Center, Isfahan University of Medical sciences, Isfahan, Iran*

**Keywords:** Acidosis, Fatty streak, Hypercholesterolemia

## Abstract

**Objective(s):**

Cardiovascular disease causes a major clinical problem in patients with end stage renal disease. Since metabolic acidosis is very common in patients with end stage renal disease, we aimed to investigate the effect of experimentally-induced metabolic acidosis on serum lipid profile and aortic fatty streak (FS) formation in normal and high-cholesterol fed rabbits.

**Materials and Methods:**

Twenty-four male rabbits were divided into four groups (n=6 each): (1) normal diet (ND): (2) hypercholesterolemic diet (HCD) (1%): (3) ND plus acidemic diet: (4) HCD plus acidemic diet. Metabolic acidosis was induced by adding 0.75% NH_4_Cl in drinking water. After 4 weeks, blood samples were taken and thoracic aortae were dissected for histological examinations.

**Results:**

Results showed that in the animals who received NH_4_Cl, metabolic acidosis was successfully induced. Serum total cholesterol and low density lipoprotein (LDL) concentrations in HCD groups were significantly higher than ND groups (*P*<0.05) and acidosis did not significantly change serum lipid levels neither in ND nor in HCD animals (*P*>0.05). Histological examination of aortae showed higher mean average grades of pathological evaluation in HCD than ND groups (2.1±0.16 vs. 0±0; *P*<0.05). Acidosis did not further increase FS formation in HCD groups (*P* >0.05).

**Conclusion:**

In this model of experimentally-induced metabolic acidosis, acidosis could not increase FS formation in HCD animals and it seems that it does not interfere in progression of atherosclerosis process.

## Introduction

Cardiovascular diseases and atherosclerosis are the leading cause of death in developed countries (1, 2). Studies indicated that the prevalence of cardiovascular disease is 20 times higher in patients with renal disease than in normal population (3) and it is one of the most important causes of death in patients with chronic renal failure (4). In addition, autopsy and clinical studies indicated higher incidence of atherosclerotic plaque in coronary arteries of patients under dialysis (5, 6).

Several studies have been done to illustrate the mechanism and cause of accelerated atherosclerosis in patients with end stage renal disease and to suggest some possible mechanisms involved in this process including endothelial dysfunction, oxidative stress, immune system disorder, high lipoprotein (a) level, hemocysteinemia and elevated tumor necrosing factor α (TNFα) and interlukin-6 (IL-6) (7-9). However, the exact mechanism is still unclear.

Metabolic acidosis is very common in patients with end stage renal disease. When clearance of creatinine is less than 30 ml/min, metabolic acidosis usually appears. There are few studies on the role of acidosis on atherosclerosis process. One study indicated that acidosis increases LDL oxidation which, in turn, involves in development of atherosclerosis (9). Atherosclerotic lesions are divided into fatty streak (FS), fibrofatty lesion, and fibrous plaque. FS is the earliest lesion and is considered as a precursor for clinical disease (10). In this study, we investigated the effect of experimentally-induced metabolic acidosis on serum lipid profile and aortic fatty streak formation in normal and high-cholesterol fed rabbits.

## Materials and Methods

***Animal groups and study design***

Male White rabbits (n=24), weighing between 1.5 and 2.5 kg were obtained from the animal careunit of Pasteur Institute of Iran. All experimental procedures were conducted in accordance with author's university guidelines. Each three animals were kept in cages at 22-25°C with 12 hr light/dark cycle and free access to standard chow and drinking water *ad libitum*. After one week habituation to animal room, blood samples were taken and the animals were randomly divided into four groups and treated by the following protocols:

(1) normal diet (ND) (n= 6); 

(2) hypercholesterolemic diet (HCD) (n= 6); 

(3) ND plus acidemic diet (n= 6);

(4) HCD plus acidemic diet (n= 6).

HCD (1%), prepared by adding one gram of pure cholesterol (Merck, Germany), was dissolved in 4 ml olive oil to 0.1 kg of commercial rabbit chow (11, 12). Acidemic diet was composed of 0.75% NH_4_Cl (Sigma Co, USA) in drinking water *ad libitum* as previously described (13). 

After 4 weeks, blood samples were taken for measurement of pH, bicarbonate and base excess (BE). The animals were, then, sacrificed with high dose of IV injection of sodium pentobarbital via the marginal ear vein and thoracic aortae were dissected for histological examinations. Body weight was measured before and after experiment.

***Serum lipid profile measurement***

Blood samples were taken before and after experiment. The samples were centrifuged and serums were kept at -70°C for further analysis. Serum total cholesterol, triglyceride (TG), and high density lipoprotein (HDL-C) were measured by calorimetric assay. Low density lipoprotein (LDL-C) was calculated with the friedwald equation.

***Histological evaluation of fatty streak formation***

After sacrifice, thoracic aortae were dissected and fixed in formalin 10% for 24 hr and then embedded in paraffin. Paraffin-embedded specimens were sectioned at 5 μm and stained by H&E. All together, 146 microscopic fields, including 720 tissue sections were obtained. The slides were evaluated by two pathologists in a double-blind manner. The specimens were graded as follows: 0: no FS formation; 1: existence of FS in 1-4 sections; 2: existences of FS in 5-9 sections; 3: existence of FS in 10-14 sections; 4: existence of FS in 15-20 sections of vessels (14). Intima to media (I/M) ratio was also measured in each sample. 

**Table 1:** Results of pH, bicarbonate and base excess in all experimental groups

	Normal diet	Normal diet + acidemic	Hypercholesterolemic	Hypercholesterolemic + acidemic
pH	7.49±0.03	7.29±.0.23^*^	7.45±0.08	7.30±0.05^**^
HCO3^-^(meq/l)	20.84±0.6	15.5±1. 1^*^	22.10±0.89	14. 6±1.24^**^
Base Excess	0.15±0.7	-7.85±2.1^*^	0.5±0.85	-9.05±1.09^**^

^*^: *P*<0.05 when compared with normal diet group; ^**^: *P* <0.05 when compared with hypercholesterolemic group

***Statistical analysis***

Data are reported as mean±SE. Data was analyzed with one-way ANOVA using Tukey's test for any differences between groups. Paired data was analyzed by paired t-test. *P* value less than 0.05 was considered as significant.

## Results

***Serum pH, bicarbonate and base excess***

Table 1 illustrates arterial pH, bicarbonate and BE after experiment. Results showed that in the animals who received NH_4_Cl for 4 weeks (groups 3&4), metabolic acidosis was induced and pH was significantly lower in those groups compare to non-treated groups (*P* <0.05). 

***Effect of metabolic acidosis on serum lipid profile***

**Figure 1:**
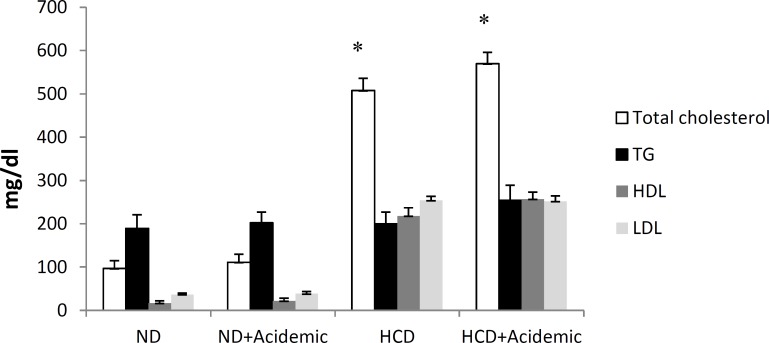
Serum lipid profile at the end of experiment in all experimental groups. *: *P* <0.05 compared to normal diet groups. ND: normal diet; HCD: high-cholesterol diet; TG: triglyceride, HDL: high-density lipoprotein; LDL: low-density lipoprotein

Body weight and serum lipid profile before experiment did not differ from one group to another. After experiment, there was no significant difference in body weight between groups. At the end of the experiment, serum total cholesterol and LDL concentrations in HCD (1%) animals were significantly higher than ND groups (*P*<0.05) (Figure 1). Acidosis did not significantly change serum lipid profile neither in normocholesterolemic nor in hypercholesterolemic animals (*P* >0.05). 

***Effect of acidosis on FS formation***

**Figure 2:**
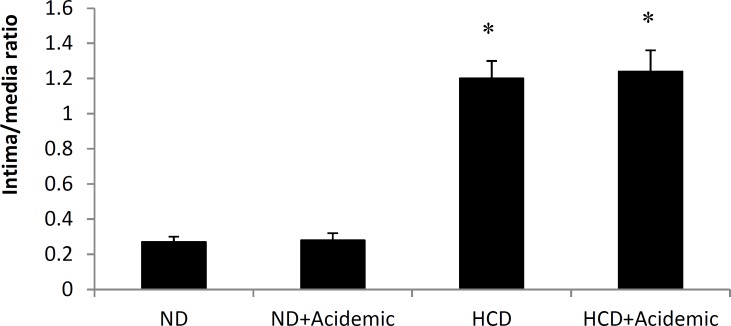
Intima to media (I/M) ratio in thoracic aortae. *: *P* <0.05 compared to normal diet groups. ND: normal diet; HCD: high-cholesterol diet

**Figure 3:**
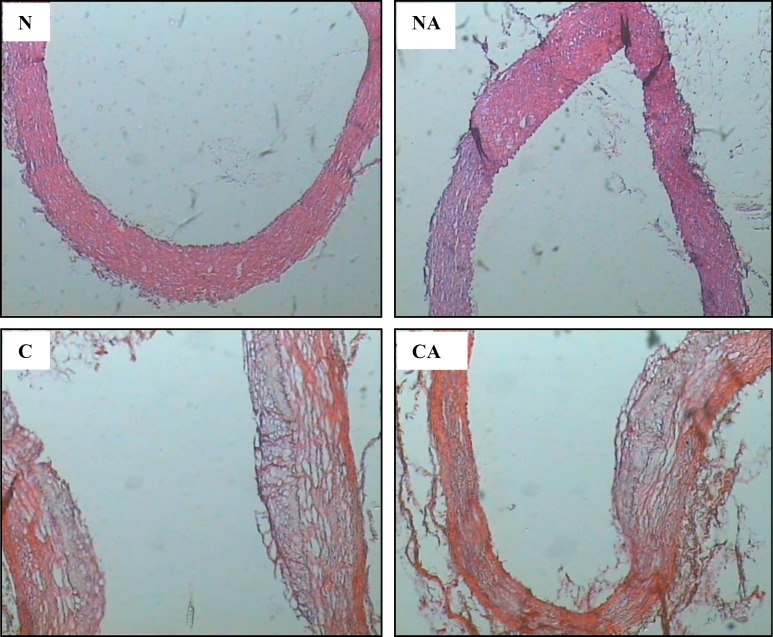
Representative photograph of thoracic aortae stained by H&E in experimental groups. N: normal diet; NA: normal diet+ acidemic; C: high-cholesterol diet; CA: high-cholesterol diet+acidemic

Histological examination of aortae showed that the mean average grades of pathological evaluation in hypercholesterolemic animals was significantly higher than normocholesterolemic groups (2.1±0.16 vs. 0±0; *P*<0.05). Acidosis did not further increase FS formation in high cholesterol diet groups (2.2±0.13 vs.2.1±0.16; *P*>0.05). I/M ratio was considerably high in HCD group compared to ND group and acidosis could not alter I/M ratio in HCD animals (Figure 2). Samples of thoracic aortae in experimental groups are shown in Figure 3.

## Discussion

Cardiovascular complications are a major cause of mortality and morbidity in patients with renal disease. Alteration of arterial function especially in large conduit arteries (15) in patients with end stage renal disease is responsible for the high incidence of cardiovascular disease (15). Studies indicated that the prevalence of atheroma in coronary arteries of patients with renal disease was approximately 30% (16). 

In this study, we found that experimentally-induced metabolic acidosis in normal and high-cholesterol fed rabbits did not alter serum lipid profile and aortic FS formation. Metabolic acidosis exists in end stage renal disease and is considered as possible mechanism for development of atherosclerosis. There are few studies on the role of metabolic acidosis on atherosclerosis. For the first time, Tavor *et al* showed that metabolic acidosis modulates interaction of aortic glycosaminoglycan with plasma LDL (13). It is also demonstrated that acidosis, by itself, can predispose LDL oxidation which is a crucial stage in atherosclerosis process (17). Therefore, macrophages can phagocyte oxidized LDL and form foam cells. FS is the earliest lesion of atherosclerosis and is considered as a precursor for clinical disease (10). In the present study, we expected the FS formation in acidemic HCD group to be higher than HCD group; however, no evidence was found to support the hypothesis that the atherosclerotic lesions increase when the acidemic diet was associated with the hypercholesterolemia.

## Conclusions

In this model of hypercholesterolemia, experimentally-induced metabolic acidosis could not increase FS formation and it seems that metabolic acidosis is not involved in progression of atherosclerosis process.
